# Microbiologic Evaluation of Colorectal Surgical Site Infections to Guide Surgical Prophylaxis Recommendations

**DOI:** 10.1017/ash.2024.317

**Published:** 2024-09-16

**Authors:** Ardath Plauche, Clare Gentry, Linda Yancey

**Affiliations:** Memorial Hermann Health System; UT Health Science Center at Houston

## Abstract

**Background:** Use of a combination of parenteral and oral antimicrobial prophylaxis prior to colorectal surgery is recommended to reduce risk of surgical site infection (SSI). Parenteral antibiotic selection is complicated by the need to target organisms likely to cause infection at the surgery site, while mitigating risk of antimicrobial resistance caused by overuse of broad spectrum agents. This study aimed to evaluate microbiologic data from colorectal surgical site infections across an 11-hospital health system. Microbiologic data from SSI events were used to assess continued appropriateness of health system standard recommendations for parenteral antibiotic prophylaxis in colorectal surgery, consisting of either cefazolin with metronidazole or cefoxitin monotherapy. **Methods:** This multicenter, retrospective, observational study was conducted from January 1, 2019 to March 31, 2023, using data extracted from the National Healthcare Safety Network (NHSN). Microbiologic data from colorectal SSIs from 2019 to 2022 were evaluated for a descriptive review of pathogen and phenotype trends. SSI data excluded patients age < 18 years, those identified as infection present at time of surgery (PATOS), or outpatient procedures. **Results:** A total of 8059 colorectal procedures were evaluated. Most SSIs were polymicrobial, with at least one pathogen detected in 65% of cases. The most commonly identified organisms were E. coli (22.5%), Enterococcus spp. (19.7%), P. aeruginosa (6.5%), Streptococcus spp. (4.9%), and C. albicans (4.7%). Change over time in antimicrobial-resistant phenotypes from 2019 to 2022 was not statistically significant for extended-spectrum cephalosporin-resistant E. coli (p=0.335), extended-spectrum cephalosporin-resistant K. oxytoca/pneumoniae (p=0.189), multi-drug resistant P. aeruginosa (0.058), methicillin-resistant S. aureus (p=0.906), or among isolates with no identified antimicrobial-resistance phenotype (p=0.096). Among E. coli, change from 2019 to 2022 in cefazolin non-susceptible, ceftriaxone susceptible isolates was not statistically significant (p=0.177). No carbapenem-resistant Enterobacterales isolates were identified among non-PATOS cases. **Conclusions:** Data does not support a change to broader spectrum agents for colorectal surgery parenteral antimicrobial prophylaxis. Continued use of cefazolin with metronidazole or cefoxitin as IV antibiotic prophylaxis in colorectal surgery is recommended, with ongoing tracking of microbiologic trends and antimicrobial susceptibility.

